# Supplementary material for: Quantitative multi-metabolite imaging of Parkinson’s disease using AI boosted molecular MRI
Source: Npj Imaging. 2025 Dec 22;3:66. doi: 10.1038/s44303-025-00130-x (PMC12722214; doi:10.1038/s44303-025-00130-x)
Supplement: Supplementary file 1 — Supplementary Information [file 44303_2025_130_MOESM1_ESM.pdf]

Supplementary information: Quantitative multi-metabolite imaging of Parkinson’s disease using AI boosted molecular MRI

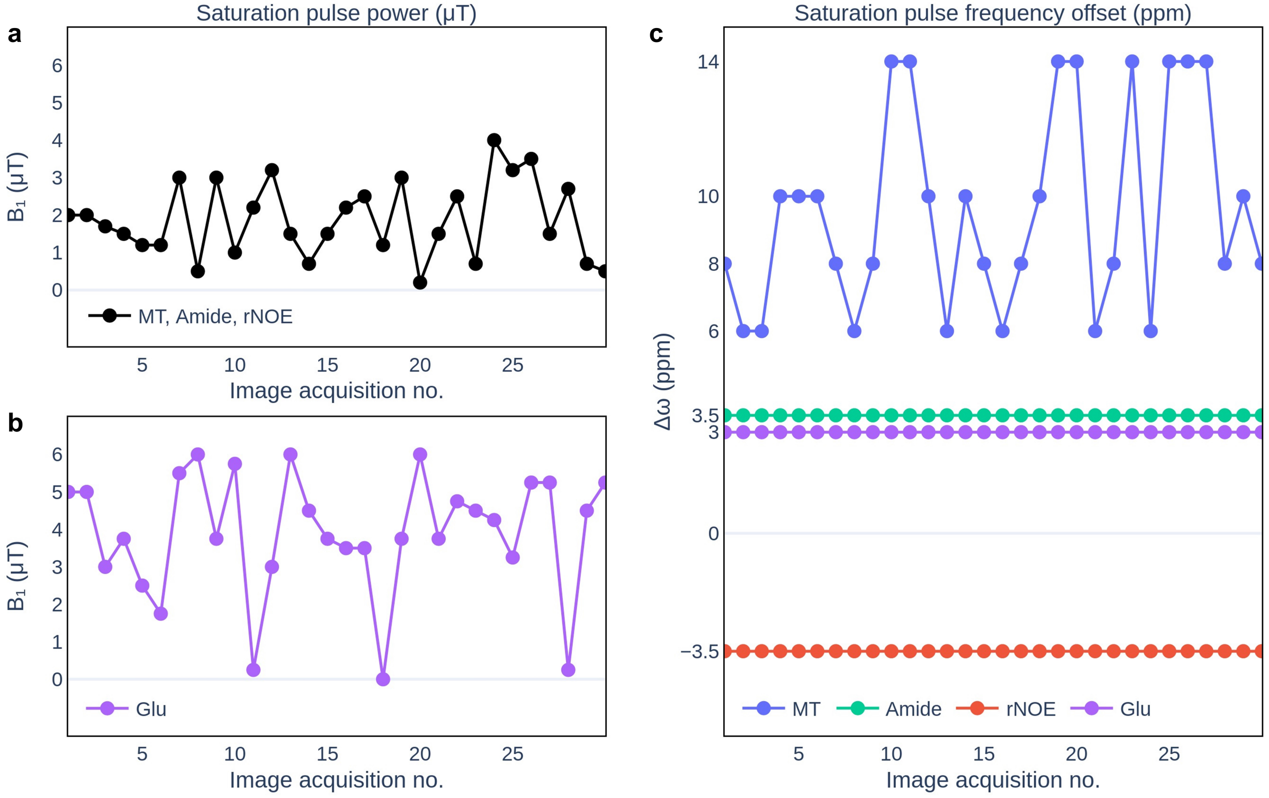

Supplementary Figure 1. Saturation pulse parameters used in the CEST MRF protocols. a. The saturation pulse power sequence used for the semisolid MT, amide and rNOE encoding protocols. **b.** The saturation pulse power sequence used for the glutamate encoding protocol. **c.** The saturation pulse frequency offsets were determined according to the target compound, namely 6-14/3.5/-3.5/3 ppm for the semisolid MT, amide, rNOE and glutamate, respectively. For the semisolid MT, amide and rNOE, the protocols had a TR/TE = 3,500/20 ms, a flip angle (FA) = 90° and a continuous wave saturation pulse duration of 2,500 ms, with total acquisition time = 120 s (including a non-saturated image acquired with TR = 15 s, not shown in the figure). The glutamate encoding protocol had (TR/TE) = 4,000/20 ms, a FA = 60°, a continuous wave saturation pulse duration of 3,000 ms and total acquisition time = 135 s (including a non-saturated image acquired with TR = 15 s, not shown in the figure).

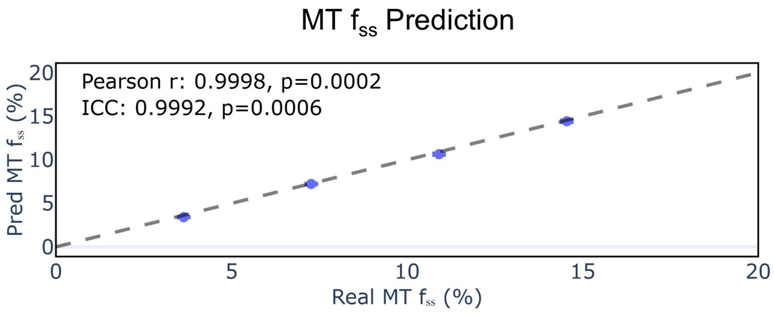

Supplementary Figure 2. Semisolid MT quantification in multi-compound digital phantoms. An excellent agreement was obtained between the MRF-based semisolid MT proton volume fractions and the ground truth (Pearson’s r = 0.9998, p = 0.0002) with high reliability (ICC = 0.9992, p = 0.0006). Data are presented as mean ± standard deviation calculated across five repetitions.

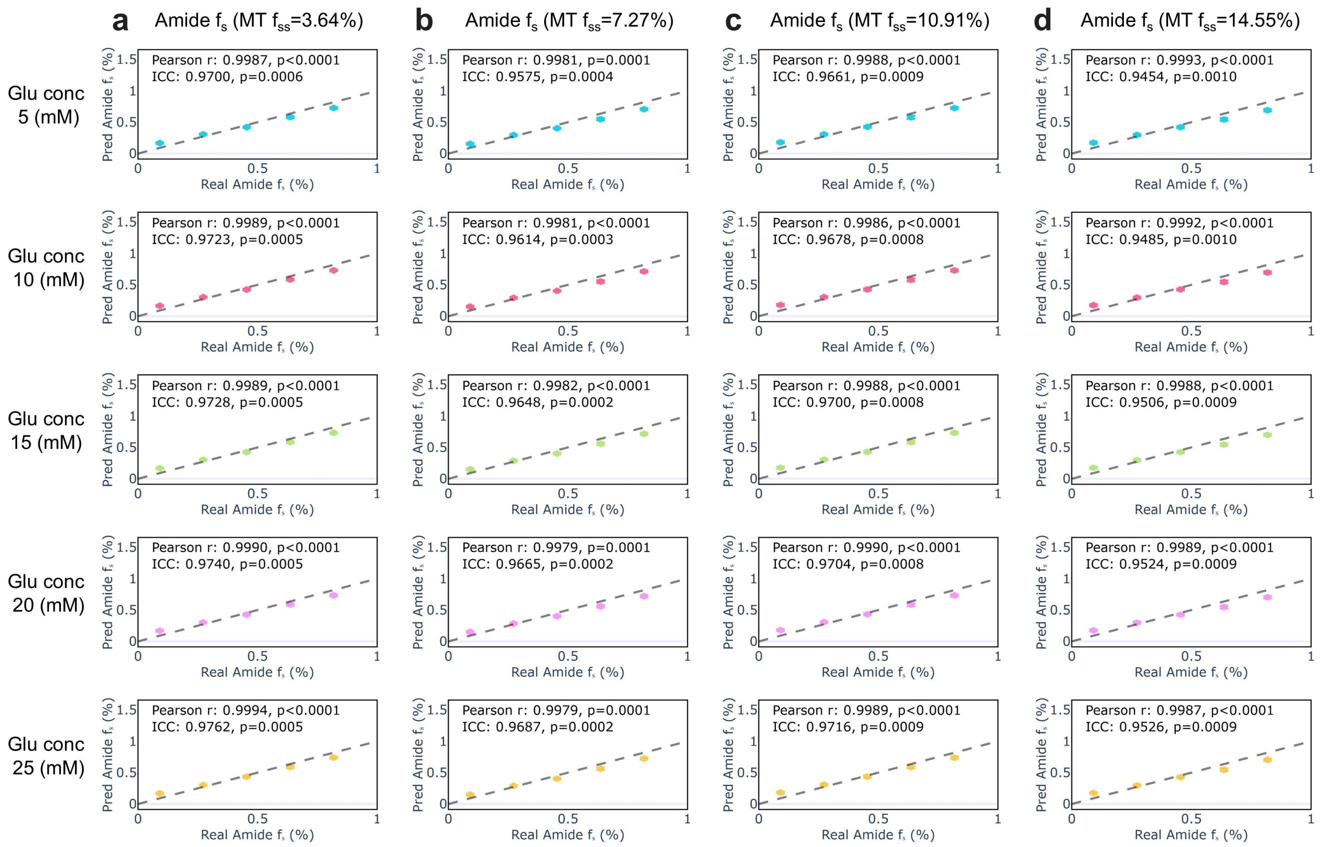

Supplementary Figure 3. Amide proton volume fraction quantification in multi-compound digital phantoms. Each column (**a-d**) represents a different semisolid MT proton volume fraction and each row represents a different glutamate concentration, as described in the top and left labels, respectively. An excellent agreement was obtained between the MRF-predicted amide proton volume fractions and the ground truth, reflected by a strong correlation (Pearson's r $\geq$ 0.9979, p $\leq$ 0.0001 across all cases) and high reliability (ICC $\geq$ 0.9454, p $\leq$ 0.0010 across all cases). Data are presented as mean ± standard deviation calculated across five repetitions.

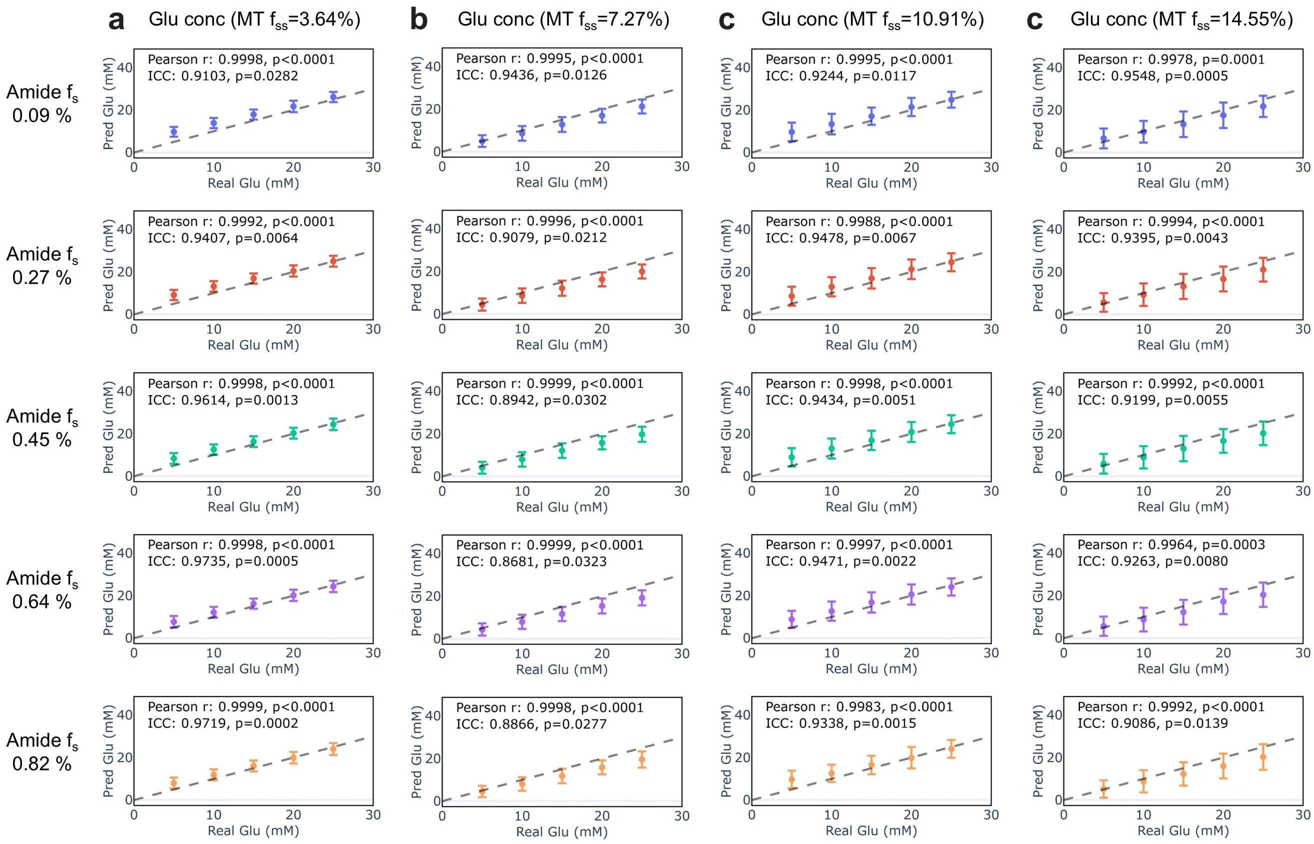

Supplementary Figure 4. Glutamate concentration quantification in multi-compound digital phantoms. Each column (**a-d**) represents a different semisolid MT proton volume fraction and each row represents a different amide proton volume fraction, as described in the top and left labels, respectively. A good agreement was obtained between the MRF-based glutamate concentrations and the ground truth, reflected by a strong correlation (Pearson's $r \geq 0.9964$, $p \leq 0.0003$ across all cases) and an $ICC \geq 0.8681$, $p \leq 0.0323$ across all cases. Data are presented as mean ± standard deviation calculated across five repetitions.

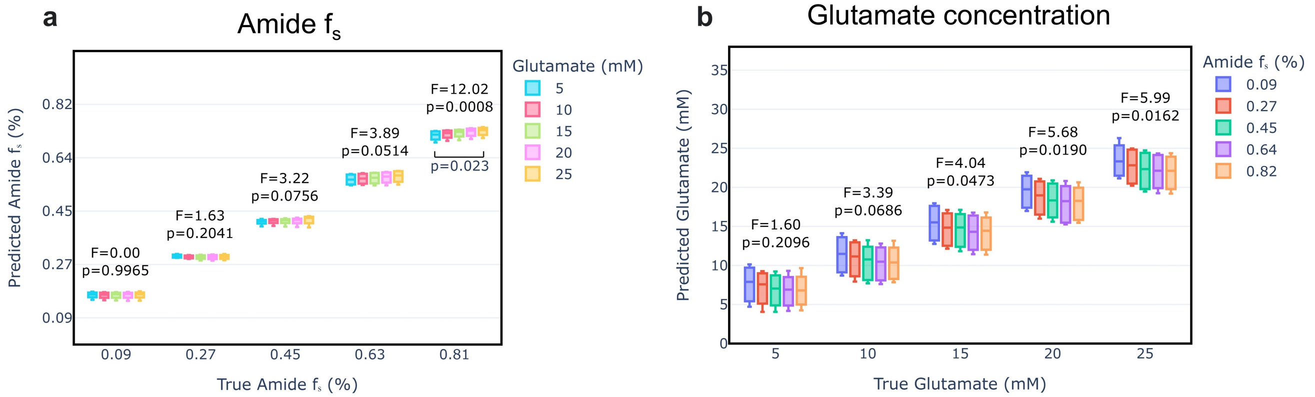

Supplementary Figure 5. Quantitative group comparison between different MRF-based predictions of amide proton volume fraction (a) and glutamate concentration (b) in multi-compound digital phantoms. One-way analysis of variance (ANOVA) was performed (see F and p-values above each five box-plot stack) followed by correction for multiple comparisons using a two-sided Tukey's multiple comparisons test (n=20 simulation measurements per boxplot, p-values shown if significant below each five box-plot stack). Notably, no statistically significant differences were obtained using Tukey's based analysis except for a single amide proton volume fraction quantification case (**a**, amide f_s = 0.81 % with Glu concentrations of 5 and 20 mM, p = 0.023). In all box plots: the central horizontal lines represent median values, box limits represent upper (third) and lower (first) quartiles, whiskers represent 1.5 x the interquartile range above and below the upper and lower quartiles, respectively.

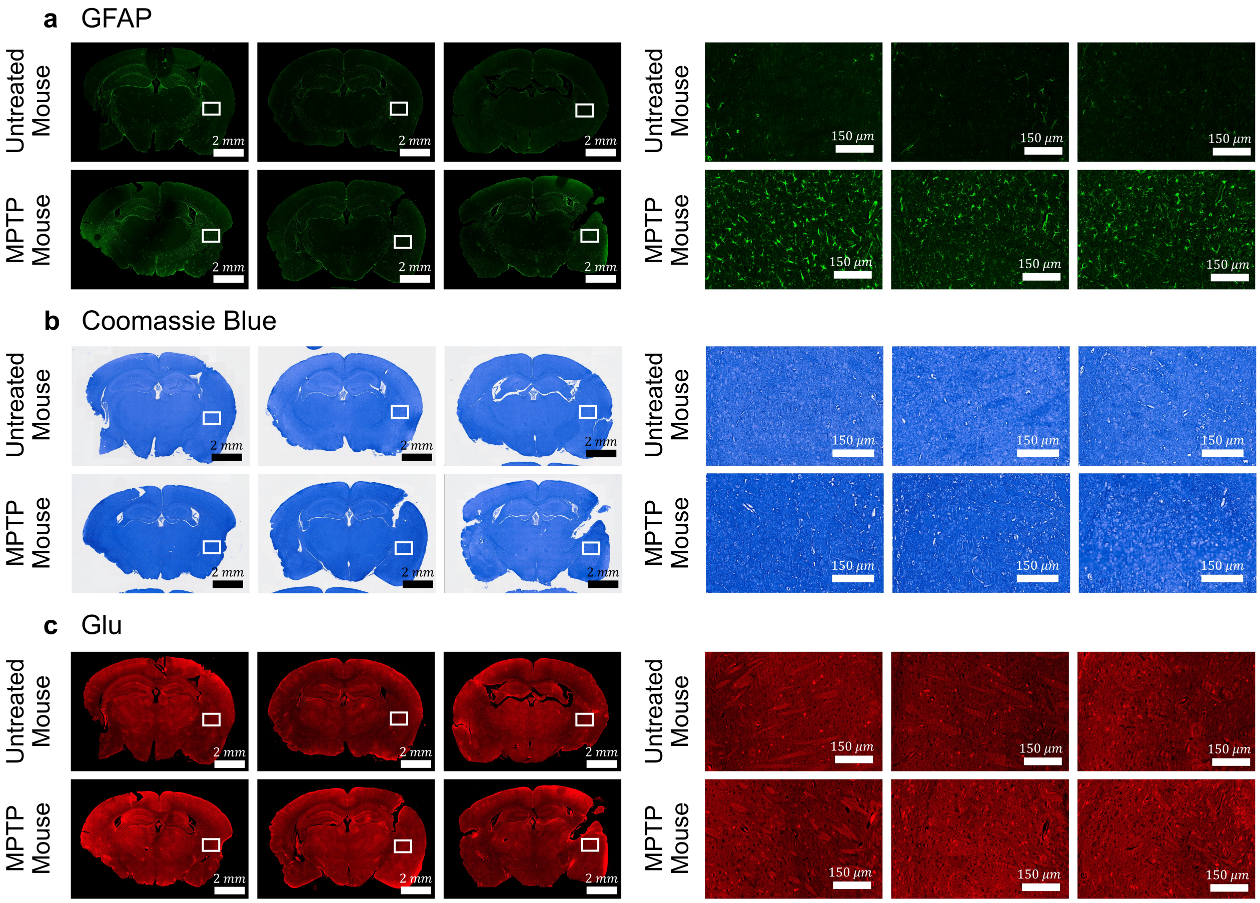

Supplementary Figure 6. Histological and Immunohistochemistry (IHC) analysis in three MPTP-treated and three untreated (control) mice. a. GFAP staining shows an increased signal in post-MPTP-treated mice. **b.** Coomassie blue shows an increase in overall protein content for MPTP-treated mice compared to control mice. **c.** A mild increase in glutamate staining signal was observed following MPTP treatment. White boxes (left) represent the magnified ROIs shown on the right panel.

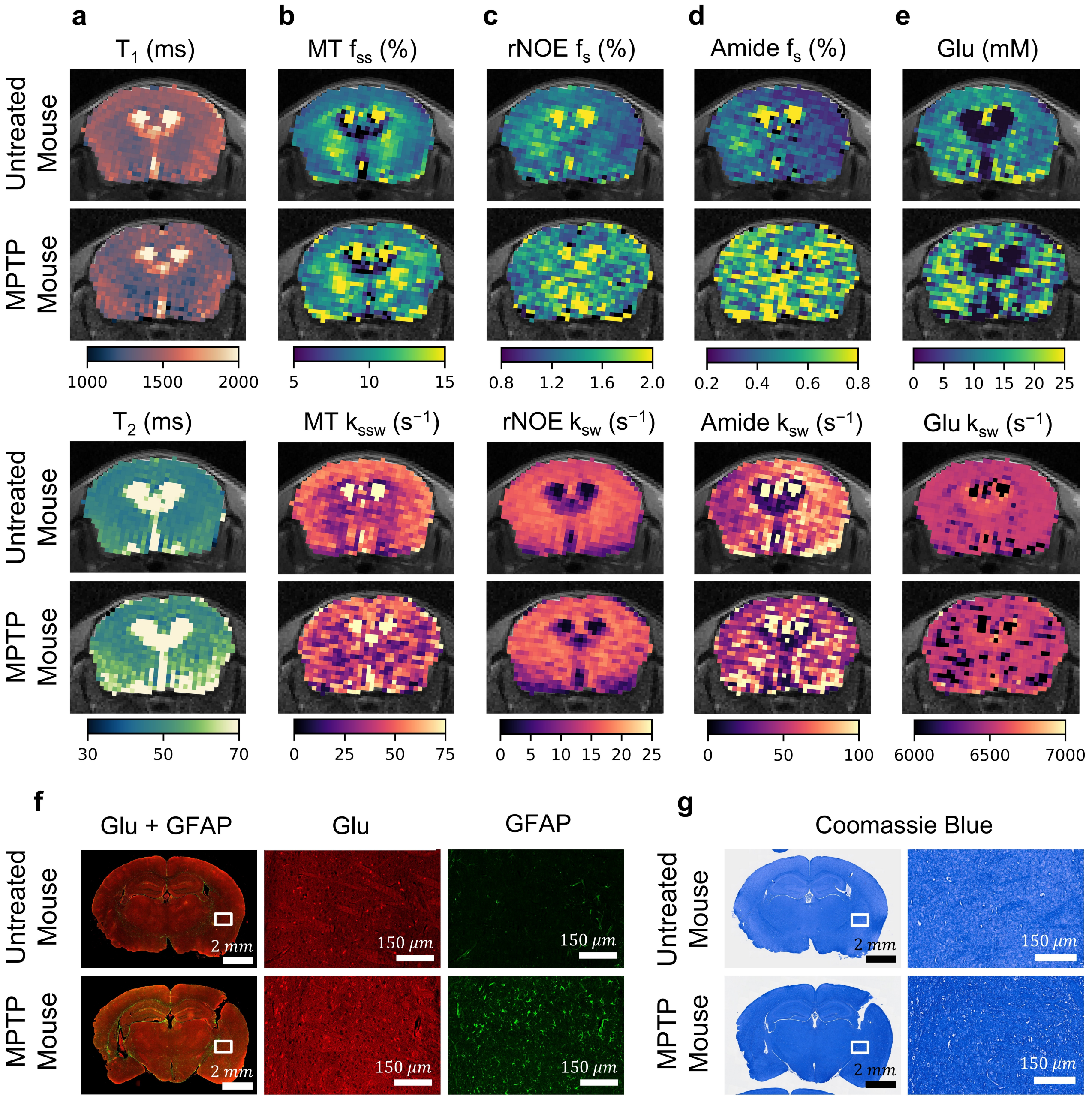

Supplementary Figure 7. A comparison between the CEST MRF parameter maps and the corresponding histological findings in an untreated (control) and an MPTP-treated mouse. a. T_1 and T_2 maps. **b**, **c**, and **d** display the proton volume fraction f_{ss}/f_s (top) and exchange rate k_{ssw}/k_{sw} (bottom) maps for the semisolid MT, rNOE and amide proton transfer pools, respectively. **e.** Glutamate concentration maps (top) and amine proton exchange rate (bottom) **f.** GFAP, glutamate, and **g.** Coomassie blue staining.

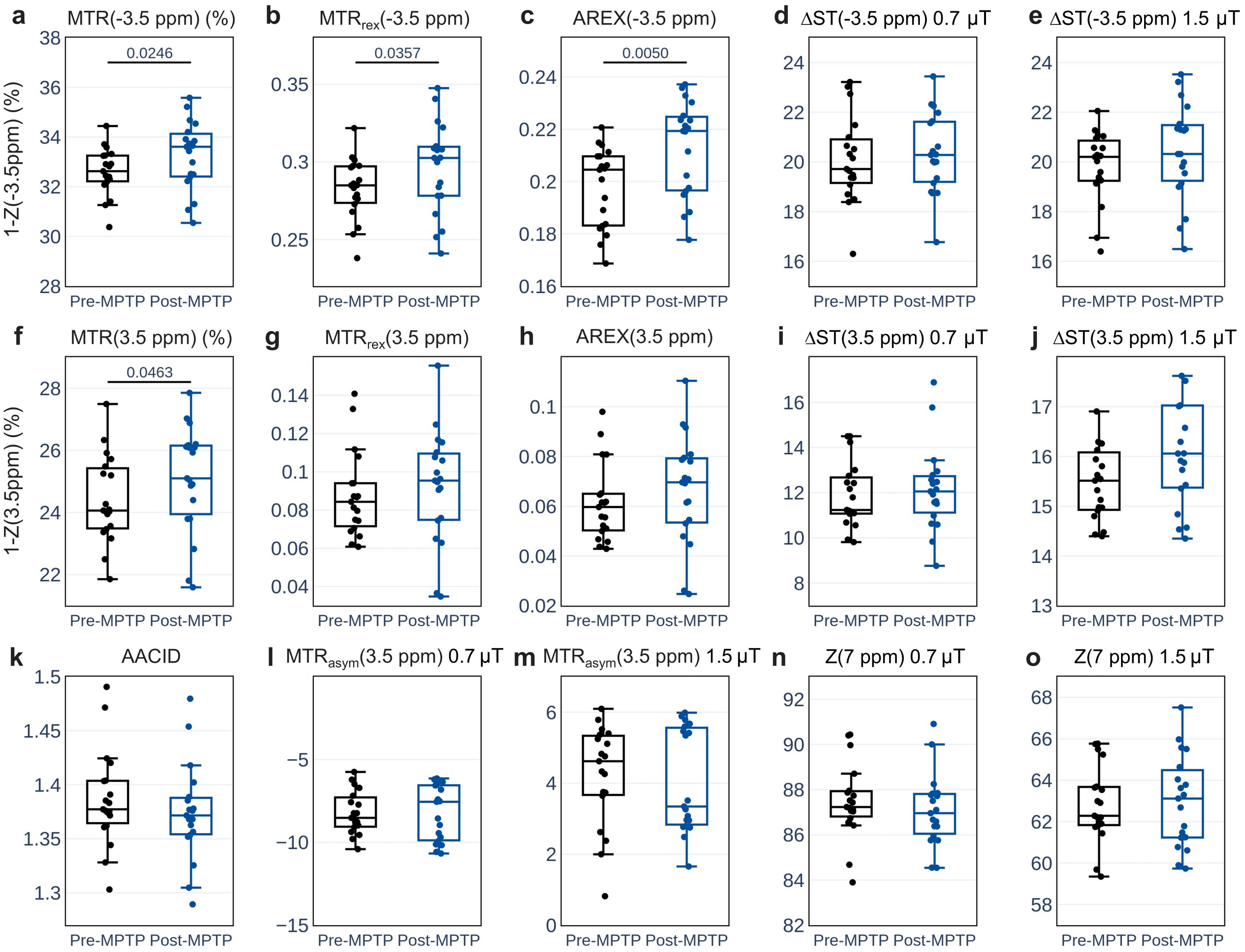

Supplementary Figure 8. Traditional MRI-based analysis using CEST-weighted imaging, performed within the same striatal ROI as used for CEST MRF. a-c. MTR(-3.5 ppm), MTR_{Rex}(-3.5 ppm) and AREX(-3.5 ppm) representing the rNOE contribution, derived from B_1 = 0.7 μT z-spectrum. **d-e.** rNOE-related observed saturation transfer difference ($\Delta ST(-3.5ppm)$) at B_1 = 0.7 μT and B_1 = 1.5 μT. **f-g.** MTR(3.5 ppm), MTR_{Rex}(3.5 ppm) and AREX(3.5 ppm) representing the amide contribution, derived from B_1 = 0.7 μT z-spectrum. **i-j.** Amide-related $\Delta ST(3.5ppm)$ at B_1 = 0.7 μT and B_1 = 1.5 μT. **k.** AACID analysis, derived from a Z-spectrum acquired using a saturation pulse power of B_1 = 1.5 μT. **l-m.** MTRasym(3.5 ppm) at B_1 = 0.7 μT and B_1 = 1.5 μT. **n-o.** MT-related Z(7 ppm) at B_1 = 0.7 μT and B_1 = 1.5 μT. In all box plots: the central horizontal lines represent median values, box limits represent upper (third) and lower (first) quartiles, whiskers represent 1.5 x the interquartile range above and below the upper and lower quartiles, respectively, and all data points are plotted.

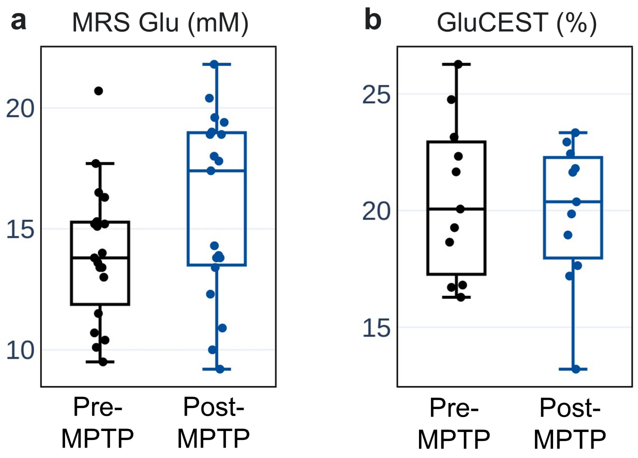

Supplementary Figure 9. Glutamate traditional MRI-based analysis using CEST-weighted imaging and ^{1}H MRS, performed within the same striatal ROI as used for CEST MRF. a. ^{1}H MRS based glutamate quantification results. **b.** GluCEST analysis. In all box plots: the central horizontal lines represent median values, box limits represent upper (third) and lower (first) quartiles, whiskers represent 1.5 x the interquartile range above and below the upper and lower quartiles, respectively, and all data points are plotted.

Supplementary Table 1. Detailed dictionary properties used for training the glutamate phantom reconstruction network. A total of 518,700 signal trajectories were generated as described below.

Glutamate Dictionary		
Water	T_1 (ms)	3,300:50:4,200*
	T_2 (ms)	1,450:50:1,900
Glu	T_1 (ms)	1,200
	T_2 (ms)	7
	$\Delta\omega$ (ppm)	3
	Labile protons	3
	f_s (mM)	1:1:30
	k_{sw} (s^{-1})	3,000:100:12,000

*The notation x:y:z represents a discrete range of values between [x, z] with y increments.

Supplementary Table 2. Detailed dictionary properties used for training the semisolid MT reconstruction network. A total of 18,522 signal trajectories were generated as described below.

MT Dictionary		
Water	T_1 (ms)	1,200:100:2,000*
	T_2 (ms)	40:10:100
MT	T_1 (ms)	Fixed to water[108]
	T_2 (ms)	0.04**
	$\Delta\omega$ (ppm)	-2.5
	f_{ss} (%)	0:1.82:23.64
	k_{ssw} (s^{-1})	0:5:100

*The notation x:y:z represents a discrete range of values between [x, z] with y increments.
** The Bloch-McConnell equations based dictionary generator yielded a Lorentzian line-shape for the semi-solid pool. To generate a linewidth equivalent to the commonly reported super-Lorentzian of 10 μs, a four times higher value (40 μs) was input to the dictionary generator[108].

Supplementary Table 3. Detailed dictionary properties used for training the rNOE (with semisolid MT background) reconstruction network. A total of 19,756,800 signal trajectories were generated as described below.

rNOE Dictionary		
Water	T_1 (ms)	1,200:100:2,300*
	T_2 (ms)	40:10:100
MT	T_1 (ms)	Fixed to water[108]
	T_2 (ms)	0.04**
	$\Delta\omega$ (ppm)	-2.5
	f_{ss} (%)	0:1.82:23.64
	k_{ssw} (s^{-1})	0:5:100
rNOE	T_1 (ms)	Fixed to water[108]
	T_2 (ms)	5
	$\Delta\omega$ (ppm)	-3.5
	f_s (%)	0.09:0.09:3.64
	k_{sw} (s^{-1})	1:1:20

*The notation x:y:z represents a discrete range of values between [x, z] with y increments.
** The Bloch-McConnell equations based dictionary generator yielded a Lorentzian line-shape for the semi-solid pool. To generate a linewidth equivalent to the commonly reported super-Lorentzian of 10 μs, a four times higher value (40 μs) was input to the dictionary generator[108].

Supplementary Table 4. Detailed dictionary properties used for training the amide/glutamate (with semisolid MT background) reconstruction network. A total of 196,465,500 signal trajectories were generated as described below.

rNOE Dictionary		
Water	T_1 (ms)	1,200:100:2,000*
	T_2 (ms)	40:10:100
MT	T_1 (ms)	Fixed to water[108]
	T_2 (ms)	0.04**
	$\Delta\omega$ (ppm)	-2.5
	f_{ss} (%)	0:1.82:23.64
	k_{ssw} (s^{-1})	0:10:100
Amide	T_1 (ms)	Fixed to water[108]
	T_2 (ms)	1
	$\Delta\omega$ (ppm)	3.5
	f_s (%)	0.09:0.09:0.82
	k_{sw} (s^{-1})	10:10:100
Glu	T_1 (ms)	Fixed to water[108]
	T_2 (ms)	7
	$\Delta\omega$ (ppm)	3
	Labile protons	3
	f_s (mM)	1:1:25
	k_{sw} (s^{-1})	5,500:250:7,500

*The notation x:y:z represents a discrete range of values between [x, z] with y increments.
** The Bloch-McConnell equations based dictionary generator yielded a Lorentzian line-shape for the semi-solid pool. To generate a linewidth equivalent to the commonly reported super-Lorentzian of 10 μs, a four times higher value (40 μs) was input to the dictionary generator[108].

108. Zaiß, M., Schmitt, B. & Bachert, P. Quantitative separation of CEST effect from magnetization transfer and spillover effects by Lorentzian-line-fit analysis of Z-spectra. J. Magn. Reson. 211, 149–155 (2011).
